# Identification, Molecular Cloning, and Functional Characterization of a Wheat UDP-Glucosyltransferase Involved in Resistance to Fusarium Head Blight and to Mycotoxin Accumulation

**DOI:** 10.3389/fpls.2018.01853

**Published:** 2018-12-13

**Authors:** Miriam Gatti, Frédéric Choulet, Catherine Macadré, Florence Guérard, Jean-Marc Seng, Thierry Langin, Marie Dufresne

**Affiliations:** ^1^Institute of Plant Sciences Paris Saclay IPS2, CNRS, INRA, Université Paris-Sud, Université Evry, Université Paris-Saclay, Orsay, France; ^2^Institute of Plant Sciences Paris-Saclay IPS2, Paris Diderot, Sorbonne Paris-Cité, Orsay, France; ^3^Unité Génétique Diversité et Ecophysiologie des Céréales INRA, UMR1095, Clermont-Ferrand, France

**Keywords:** wheat, Brachypodium, synteny analysis, UDP-glucosyltransferase, Fusarium head blight (FHB), deoxynivalenol (DON)

## Abstract

Plant uridine diphosphate (UDP)-glucosyltransferases (UGT) catalyze the glucosylation of xenobiotic, endogenous substrates and phytotoxic agents produced by pathogens such as mycotoxins. The *Bradi5g03300* UGT-encoding gene from the model plant *Brachypodium distachyon* was previously shown to confer tolerance to the mycotoxin deoxynivalenol (DON) through glucosylation into DON 3-*O*-glucose (D3G). This gene was shown to be involved in early establishment of quantitative resistance to Fusarium Head Blight, a major disease of small-grain cereals. In the present work, using a translational biology approach, we identified and characterized a wheat candidate gene, *Traes_2BS_14CA35D5D*, orthologous to *Bradi5g03300* on the short arm of chromosome 2B of bread wheat (*Triticum aestivum L*.). We showed that this UGT-encoding gene was highly inducible upon infection by a DON-producing *Fusarium graminearum* strain while not induced upon infection by a strain unable to produce DON. Transformation of this wheat UGT-encoding gene into *B. distachyon* revealed its ability to confer FHB resistance and root tolerance to DON as well as to potentially conjugate DON into D3G *in planta* and its impact on total DON reduction. In conclusion, we provide a UGT-encoding candidate gene to include in selection process for FHB resistance.

## Introduction

Wheats comprising bread wheat (*T. aestivum*) and durum wheat (*T. turgidum* ssp. *durum*) are among the major cereals produced worldwide, presently raking second after maize (http://www.fao.org/faostat/en/) However as other major crops wheat is confronted to different biotic stresses during its development, inducing deteriorations in both yield and grain quality. Common in European bread wheat varieties, Fusarium Head Blight, also named wheat scab, is one of the most important diseases of cereal crops (Dweba et al., 2017). *Fusarium graminearum* (*Gibberella zeae*, teleomorph) is the main causal agent, commonly infecting barley (*Hordeum vulgare*), bread wheat (*T. aestivum*), durum wheat (*T. durum*), and oat (*Avena sativa*) (Walter et al., 2010). Effects are yield loss, diminution in seeds and contamination with deoxynivalenol (DON) and other trichothecenes (D'Mello et al., 1999). DON belongs to type B trichothecenes (TCTB), it is a sesquiterpene molecule (Pestka, 2007; Sobrova et al., 2010) able to inhibit protein synthesis and implicated in the modification of Krebs cycle and polyamine biosynthesis. Because of its toxicity for humans and animals, tolerated levels have been established in Europe (Knutsen et al., 2017). The biosynthetic pathway for DON production has been previously described (Yazar and Omurtag, 2008), in particular, using mutant strains of *F. graminearum* (Alexander et al., 2011). DON-negative mutant strains impaired in the *Tri5* gene coding for the trichodiene synthase, the first enzyme of the biosynthetic pathway, are unable to efficiently colonize wheat spikes (Bai et al., 2002; Jansen et al., 2005; Maier et al., 2006). DON production seems not to be necessary for fungal pathogenicity, but essential for efficient spikes and seeds colonization. Hence, DON may act also as an aggressiveness factor in wheat (Mesterhazy, 1995). Wheat scab resistance is a polygenic inherited trait. Numerous QTLs have been identified in *T. aestivum* located all over the genome (Buerstmayr et al., 2009; Samad-Zamini et al., 2017). One of the major quantitative trait loci in wheat is *Fhb1* (*Qfhs.ndsu-3BS*), involved in disease spread along the spike, also referred to as type II resistance, as is the case for numerous major QTLs (Buerstmayr et al., 2009). Lemmens et al. (2005) showed a correlation between the presence of this QTL and metabolization of DON into DON-3-*O*-glucoside. Metabolic profiling revealed that DON can be detoxified through glucosylation and *S*-glutathionylation (Berthiller et al., 2009; Kluger et al., 2013, 2015; Warth et al., 2015).

Glucosyltransferases (UGTs) are a multigenic superfamily of enzymes found in all living organisms, in particular in plants (Keegstra and Raikhel, 2001). Plant family 1 UGTs, also referred to as secondary metabolism UGTs have various functions, including to the detoxification of exogenous compounds such as organic pollutants, secondary metabolites produced by fungi (including mycotoxins) and herbicides that can represent a danger for plants (Messner et al., 2003; Gachon et al., 2005). Despite the identification of a number of UGT-encoding genes induced by *Fusarium* infection or DON application in transcriptomic studies (Boddu et al., 2006; Gardiner et al., 2010; Lulin et al., 2010; Schweiger et al., 2010, 2013a,b; Nussbaumer et al., 2015), few plant UGTs able to glucosylate DON have been characterized in cereals (Li et al., 2015; Pasquet et al., 2016; Xing et al., 2018; Zhao et al., 2018). Most functional analyses were conducted *in vitro* through the use of a heterologous system such as yeast or *Escherichia coli* (Schweiger et al., 2010, 2013a,b) or *Arabidopsis thaliana* (Shin et al., 2012).

In the last few years, *B. distachyon* has emerged as a new model grass for cereals (Opanowicz et al., 2008; Brkljacic et al., 2011; Fitzgerald et al., 2015). It has a small (272 Mbp) diploid and fully sequenced genome (The International Brachypodium Initiative, 2010), as well as a number of genetic and genomic tools extremely useful for functional genomics studies. Furthermore, the high level of synteny between the genomes of *Brachypodium* and wheat has already been used to identify orthologous relationships (Kumar et al., 2009). Transcriptomic analyses of the interaction between *B. distachyon* (Bd-21 ecotype) and *F graminearum* strains able or not to produce DON showed the induction of 6 *UGT* genes, organized in cluster on chromosome 5 of the *B. distachyon* genome (Schweiger et al., 2013a; Pasquet et al., 2014) among which *Bradi5g03300* is the only gene able to confer strong tolerance to DON through glucosylation of DON into DON 3*-O*-glucose in yeast (Schweiger et al., 2013a). Functional analyses *in planta* of this gene showed increased sensitivity of mutant lines to the toxin and increased susceptibility to the fungal pathogen (Pasquet et al., 2016). Conversely, the overexpressor lines showed a tolerance to the toxin and quantitative resistance to *F. graminearum* (Pasquet et al., 2016). The goals of the present work were to identify wheat candidate genes orthologous to the *B. distachyon Bradi5g03300* gene using a synteny-based approach and to determine their expression pattern following fungal infection. An identified wheat ortholog was further introduced by genetic transformation into *B. distachyon* Bd21-3 ecotype to rapidly determine its ability to conjugate DON into D3G *in planta* and its relationship with FHB resistance.

## Materials and Methods

### Plant Material and Growth Conditions

*Brachypodium distachyon* wild type Bd21-3 and transgenic lines were cultivated in a growth chamber under 20 h (h) light period at 23 ± 5°C under fluorescent light (265 μE m^−2^ s^−1^ at the soil level). The spring wheat variety Apogee was cultivated in a growth chamber under 16 h light period at 24 ± 5°C under fluorescent light (265 μE m^−2^ s^−1^ at the soil level). Seeds were surface sterilized by incubation in 0.6% sodium hypochlorite for 3 min (wheat) or 5 min (*B. distachyon*) with gentle shaking followed by three rinses for 10 min in sterile distilled water and incubated for 4 days at 4°C in the dark. Plants were grow on 2:1 mixture of compost (Tref terreau P1, Jiffy France SARL, Trevoux, France) and standard perlite (Sinclair, Gainsborough,UK), soaked with an aqueous solution containing a carbamate fungicide (Previcur® at 2 mL/L, Bayer Crop Sciences, Lyon, France) and a larvicide (Hortigard® at 1 g/L, Syngenta France, Guyancourt, France). Plants were watered in 3 days intervals using a standard nutritional solution.

### *In vitro* Seed Germination and Roots Development of *Brachypodium distachyon*

For root tests *in vitro* (segregation and root tolerance) the palea and lemma of each seed were removed and seeds were surface sterilized as previously described. Surface-sterilized seeds were incubated at 4°C for 5 days. Consequently, the sterilized *Brachypodium* grains were sown on Murashige and Skoog (MS) with 3% saccharose and vitamins (100 mg/L of myo-inositol and 0.1 mg/L of thiamin-HCl) in squared petri dishes (12^*^12 cm). To study root tolerance to DON in *Brachypodium* transgenic lines, 50 μM DON (Sigma-Aldrich, Lyon, France) was added to the medium. These experiments were performed in four biological replicates with at least 30 independent seeds per replicate. To test for the segregation of the transgene, the medium contained 40 mg L^−1^ hygromicin. Plates were incubated for 48 h in the dark at 24°C. The box is moved under 16 h light period at 24 ± 5°C under fluorescent light (265 μE m^−2^ s^−1^ at the soil level). Measurements were made after 7 days growth.

### *Fusarium graminearum* Strains, Maintenance and Spore Production

*Fusarium graminearum* strain *Fg*DON^+^ (PH-1, with a DON/15-ADON chemotype; Cuomo et al., 2007) was maintained on potato dextrose agar (PDA) plates. To obtain fungal spores, 2-to 4-mm2 plugs from 15-days (d)-old PDA plates were inoculated in liquid mung bean medium (Bai and Shaner, 1996; 10 plugs for 20 mL) and incubated at 150 rpm at room temperature for 5–6 d. Fresh mung bean medium was then inoculated at 1/10 by the first sporulation and incubation was renewed under the same conditions. For pathogenicity assays, spores were filtrated onto sterile Miracloth (Calbiochem) and resuspended in 0.01% Tween 20 at a final concentration of 10^5^ spores.mL^−1^.

### Pathogenicity Assays

Inoculation was performed by pipeting 300 spores (3 μL of a 10^5^ spores.mL^−1^ spore suspension) of the *Fg*DON^+^ strain into a central floral cavity of the second spikelet left from the top at mid-anthesis (32 days after sowing) in case of *B. distachyon*. For wheat, the same protocol was applied but 500 spores (5 μL of 10^5^ spores.mL^−1^ suspension) were brought into a central flower of secondary spikes at BBCH65 stage (56 days after sowing). For both plant species, the first 24 h, inoculated heads were kept in the dark then incubated with a photoperiod of 8 h of light and 16 h of darkness at 20 ± 2°C with the same light intensity used for the plant development. Application of 0.01% Tween 20 was used as a control. For point inoculation, symptoms were evaluated 7 and 14 days after inoculation (dai) with a scoring scale ranging from 0 (no symptoms) to 4 (spikelet adjacent to the inoculated spikelet fully symptomatic) (Pasquet et al., 2016). Pathogenicity assays all performed in three independent biological replicates.

### DNA Extraction and Fungal DNA Quantification by qPCR

For quantification of *F. graminearum* DNA in *B. distachyon*, 5 spikes spray-inoculated with *Fg*DON^+^ strain were pooled per plant genotype 14 days after inoculation. Three independent biological replicates were performed. Whole genomic DNA was extracted using the protocole set up by Atoui et al. (2012). Quantification of fungal DNA was performed by quantitative PCR (qPCR) on 10 ng of total DNA using primers specific for the fungal 18S ribosomal subunit-encoding genomic region (Mudge et al., 2006). Reactions were performed in a LightCycler LC480 real-time PCR system (Roche Diagnostics, France). The absolute quantity of fungal DNA in each sample was determined by comparing with a standard curve corresponding to serial dilutions of fungal genomic DNA alone.

### RNA Extraction and cDNA Synthesis

Leaves, roots and spikes from 4-week-old independent plants or from infected spikes were ground in liquid nitrogen, and total RNA was extracted from 0.1 g of the resulting powder using Trizol (Invitrogen, Life Tech) followed by an RNase-free DNAse I step (Ambion, applied Biosystems) according to the manufacturers' instructions. cDNA synthesis was performed on 1 μg of total RNA using the ImProm-II reverse transcription system (Promega). The product was diluted 10 times in nuclease-free water.

### RT-qPCR Primer Wheat Design and Validation

Using the software SPADS (Specific Primers and Amplicon Design Software, saruman.versailles.inra.fr) primers were designed for a RT-qPCR application: an amplicon size between 60 and 150 bp, a melting temperature between 55 and 62°C and a percentage of GC between 30 and 80%. The selected primers (Supplementary Table 1) were validated using nullisomic lines and cloning technology (Supplementary Figure 1).

### Quantitative Reverse Transcription-PCR (RT-qPCR)

RT-qPCR was performed on 2 μL of the diluted cDNA product using 8 pmoles of each specific primer and 10 μL of SYBRGreen Master Mix in a final volume of 20 μL. Reactions were performed in a LightCycler LC480 real-time PCR system (Roche Diagnostics, France). All RT-qPCR were carried out on three independent biological replicates, each in technical duplicate. The comparative Ct method was used to evaluate the relative quantities of each amplified product in the samples. The Ct was automatically determined for each reaction by the Light Cycler LC480 real-time PCR systems with default parameters. The specificity of the RT-qPCR was determined by melt-curve analysis of the amplified products using the standard method installed in the system. RT-qPCR was performed using SYBR Green detection, at each run with primer pairs specific for different candidate *UGT* genes and two housekeeping genes (*ACT* and *TUB*, see Supplementary Table 1, Hong et al., 2008). Relative quantification was performed to determine the changes in steady-state mRNA levels of the different genes by comparing to the levels of two internal control genes (*ACT* and *TUB*). To calculate the expression of the genes of interest we chose to set their expression relatively to the two reference genes based on threshold values (Ct) using the ΔΔCt method. In addition ratio of the wheat transgene expression in *B. distachyon* was calculated using: Ct (transgene)/Ct (control gene, *UBC18*) ^*^100.

### *In silico* Analysis

A synteny-based approach was applied using the *B. distachyon* annotated genome sequence (The International Brachypodium Initiative, 2010) and the bread wheat IWGSC CSS (chromosome survey sequence) annotation v2.2 (International Wheat Genome Sequencing International Wheat Genome Sequencing Consortium (IWGSC), 2014). BLAST (Altschul et al., 1990) was used to perform similarity-search of protein sequences of *Brachypodium* genes against all known wheat deduced protein sequences (IWGSC CSS annotation v2.2). Hits were filtered by considering only the 552 genes that showed an alignment with at least 40% amino-acid identity covering at least 50% of the length of the query sequence. Wheat homeologous genes were further identified using BLAST (Altschul et al., 1990) with a threshold of 90% minimum identity. The phylogenetic analysis was conducted using MEGA version 7 (Kumar et al., 2016) using CLUSTALW (Thompson et al., 1994) for multiple protein sequence alignment and the Maximum Likelihood method based on the JTT matrix-based model (Jones et al., 1992). Sequences for markers harboring QTLs for Fusarium Head Blight resistance were obtained from the GrainGenes database and a BLAST analysis was developed to localize sequences on chromosomes using Triticum_aestivum_CS42_TGAC_v1 assembly.

### Construction and Transformation

The *Traes_2BS_14CA35D5D* cDNA was amplified from cDNAs synthetized from infected spikelets of the wheat variety Apogee using primers 5′-CGCGGATCCGCGATGGAGAGCACGGG-3′ and 5′-GGATATCCTCAAATTGACGAATACTTGGCAGC-3′ adding a *Bam*HI and an *Eco*RV restriction site (underlined). The PCR product was then digested using the *Bam*HI and *Eco*RV restriction enzymes, purified using a NucleoSpin Gel and PCR clean-up kit (Macherey-Nagel, France), and then ligated into the pENTR1A Gateway® plasmid linearized by the same restriction enzymes. The resulting construct was used to transfer the *Traes_2BS_14CA35D5D.1* cDNA into the pIKb002 binary vector (Himmelbach et al., 2007) by *in vitro* recombination using the Gateway® LR Clonase II Enzyme mix (InVitrogen, France), allowing expression of the wheat cDNA under the control of the strong and constitutive promoter of the *Zea mays Ubi1* gene. The pIKb002: *Traes_2BS_14CA35D5D.1* binary vector (Supplementary Figure 2A) was electroporated into *Agrobacterium tumefaciens* AGL1 electrocompetent cells. Embryogenic calli of *Brachypodium* were transformed by *A. tumefaciens*-mediated transformation (as described in Vogel and Hill, 2008). Two independent homozygous plants were shown to contain and express the construction and a selection of transformants in MS agar medium (Murashige and Skoog, 1962) containing 40 mg.L^−1^ hygromycin B was developed to verify that the two independent transformants corresponded to single-locus insertion lines at the T2 generation.

### Mycotoxin Extraction and Analysis

Five-hundred milligrams of fresh ground material (spikes or spikelets infected by the FgDON+ strain) was extracted with 7 mL of acetonitrile/water (84/16, v/v) for 1 h at room temperature on a tube rotator (50 rpm). Before extraction, 0.5 μg of fusarenon X was added in each sample as an internal standard. After centrifugation (5 min at 5,000 g), the supernatant was purified on Trichotecene P Columns (P51 R-Biopharm) and 3 ml of filtrate were evaporated at 50°C in a dryness of nitrogen. The pellet was resuspended in 400 μl of methanol/water (50/50, v/v) and filtered through a 0.20 μM filter before analysis. DON, DON-3-O-glucose, 15-ADON, and fusarenon X concentrations were determined using HPLC-MS/MS analyses. These analyses were performed using a micro TOF II (Brukner) equipped with an Acquity UPLC (Waters), a reverse phase Kinetex™ 1.7 μm XB-C18 column (100 × 2.1 mm, Phenomenex) maintained at 45°C and a ESI source Solvent A consisted of methanol/water (10/90, v/v) and solvent B consisted of methanol / water (90/10, v/v). The flow rate was kept at 400 μL/min and went to the electrospray source. Gradient elution was performed with the following conditions: 2 min with a linear gradient from 80 to 5% A, 2 min held at 5% A, 1 min linear gradient from 5 to 80% A and 80% A for 2 min postrun reconditioning. The injection volume was 5 μL. The electrospray interface was used in the negative ion mode at 195°C with the following settings: nebulizer gas, 4 bar.; dry gas, 8.5 L/min; ion spray voltage, −3,700 V. Quantification was performed using external calibration with DON (Sigma-Aldrich, Lyon, France), DON-3-O-glucose (Sigma-Aldrich, Lyon, France), 15-ADON (Sigma-Aldrich, Lyon, France), and fusarenon X standard solutions, ranging from 10 to 1,000 ng/mL.

## Results

### Identification of Potential Wheat Orthologs of the *B. distachyon Bradi5g03300* Gene

A macrosynteny-based approach was first used to identify the wheat genomic regions orthologous to the region of *B. distachyon* chromosome 5 carrying the *Bradi5g03300*. A 13 Mb region encompassing *Bradi5g03300* was considered, from *Bradi5g00230.1* to *Bradi5g09987.1*, which contained 708 gene models. We used BLAST (Altschul et al., 1990) to perform similarity-search of the protein sequences of *Brachypodium* genes against all known wheat deduced protein sequences (IWGSC CSS annotation v2.2). This revealed the majority (386, 70%) of these filtered *B. distachyon* genes has a best BLAST hit on wheat chromosome 2A, 2B, or 2D (Supplementary Table 2). This confirmed that the region carrying *Bradi5g03300* is syntenic with a region of the short arms of group 2 chromosomes in wheat.

**Figure 1:**
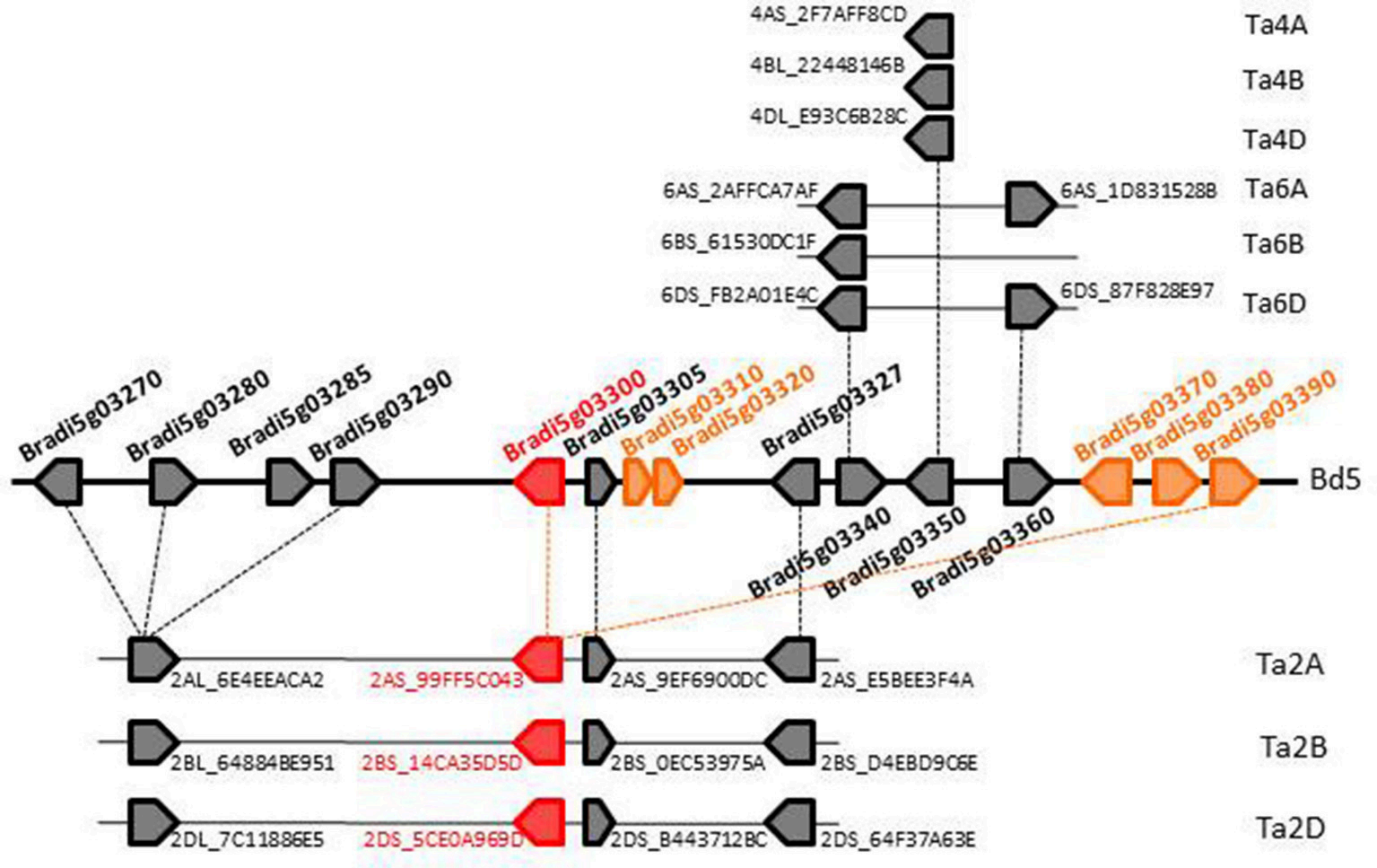
Comparative genomic organization of the UDP-glycosyltransferases (UGT) gene cluster in *Brachypodium distachyon* and the syntenic region in wheat. Wheat genomic regions syntenic with the *Brachypodium* locus carrying the *Bradi5g03300* gene correspond to a locus on the short arm of chromosomes 2 (2AS, 2BS, 2DS). For convenient display, we removed the prefix “Traes_” from the wheat gene names. Bd, *B. distachyon*; Ta, *T. aestivum*.

To go further, analysis of the micro-colinearity was carried out for region *Bradi5g03270.03390* (13 genes; Figure 1). Among these 13 genes, six are paralogous and belong to the *UGT* family: *Bradi5g03300-3310-3320* and *Bradi5g03370-3380-3390*, comprising a 50 kb-cluster of tandemly duplicated genes. The flanking gene models *Bradi5g03310* and *03320* are both truncated and correspond in fact to the 5′ and 3′ of a complete *UGT* gene, respectively, suggesting they are the two parts of a single gene. Analyses conducted on the *T. aestivum* genome led to the identification of wheat genes syntenic with *B. distachyon* genes around the *Bradi5g03300* gene on wheat chromosomes 2A, 2B, and 2D (Figure 1). Concerning *Bradi5g03300*, highest sequence identity was found with wheat homeologous triplet *Traes_2AS_99FF5CO43* (Accession No. MK166043)*, Traes_2BS_14CA35D5D* (Accession No. MK166044), and *Traes_2DS_5CE0A969D* (Accession No. MK166045). Additional hits sharing lower identity were also detected on wheat chromosomes 5 and with other genes on chromosome 2, showing a complex evolutionary history with rounds of gene duplications (Supplementary Table 2).

Copies of genes on chromosomes 2AS (*Traes_2AS_99FF5CO43*) and 2DS (*Traes_2DS_5CEOA969D*) are likely truncated in their 5′ end due to their position close to contig ends and the deduced partial UGT proteins exhibit 245 and 266 amino acids, respectively, while the length of complete protein deduced from the *Traes_2BS_14CA35D5D.1* is 462 amino acids (Supplementary Presentation 1). All three putative proteins contain in their C-terminus the Plant Secondary Product Glycosyltransferase (PSPG) box consensus sequence characteristic for family 1 UGTs (Gachon et al., 2005), constituted of a stretch of 44 amino acids. Among the 245 amino acids in common to the three proteins Traes_2AS_99FF5CO43.1, Traes_2BS_14CA35D5D.1, and Traes_2DS_5CEOA969D.1, sequence identity ranges from 83% (2A−2D) to 89% (2A−2B) and similarity from 89% (2A−2D) to 91% (2A−2B, 2B−2D; Supplementary Presentation 1).

**Figure 2:**
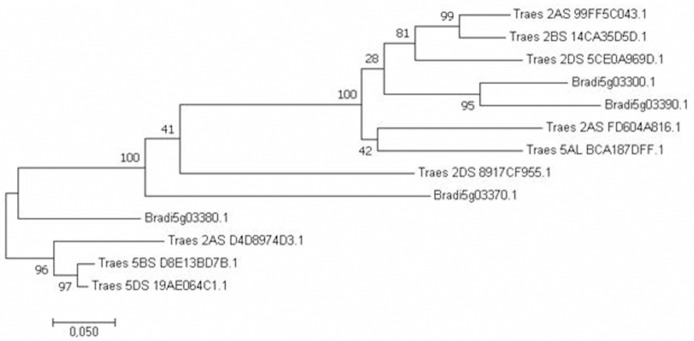
Phylogenetic analysis of the putative wheat candidate UGT proteins. The upper clade contains UGTs corresponding to putative wheat orthologs of the *B. distachyon* Bradi5g03300 UGT, encoded by the *Traes_2BS_14CA35D5D* gene and its homeologous genes *Traes_2AS_99FF5CO43* and *Traes_2DS_5CEOA969D*. A 225-AA sequence corresponding to the C-terminal part of all proteins was used to generate the multiple sequence alignment (SeaView Version 4.6.1). Phylogenetic tree was developed using Jalview 2.10.2b1, Neighbor-Joining, Blosum 62 matrix.

We then built a phylogenetic tree based on a multiple alignment of wheat protein sequences (IGWSC CSS v2 assembly for *Triticum aestivum*) that are homologous to the Bradi5g03300 protein. Only 9 wheat proteins with a BLASTP score higher than 400 were considered here, encoded by genes located on wheat chromosomes 2 (including the three homeologs identified in the microsynteny analysis) and chromosomes 5 which were designated on the phylogenetic tree after the corresponding gene accession numbers. Wheat protein sequences were aligned with *B. distachyon* UGT protein sequences already identified as tightly clustered on *B. distachyon* chromosome 5 (Schweiger et al., 2013a), including the DON-detoxifying UGT Bradi5g03300 but also the Bradi5g03370, Bradi5g03380, and Bradi5g03390 proteins. Due to truncated wheat sequences (partially assembled), only 225 amino-acids corresponding to the C-terminal part of all proteins, containing the PSPG box, were used for multiple protein sequence alignment to generate a tree using the Maximum Likelihood method. As evidenced on Figure 2, apart from two wheat proteins more distantly related to the others (Traes_2BS_D89C56A09.1 and Traes_2AS_FD604A816.1), wheat and Brachypodium UGT proteins mainly fall into two clades. One clade contains the two *B. distachyon* Bradi5g03300 and Bradi5g003390 proteins and wheat proteins encoded by the three homeologs previously identified: *Traes_2AS_99FF5CO43, Traes_2BS_14CA35D5D*, and *Traes_2DS_5CEOA969D*.

### *Traes_2BS_14CA35D5D* Potentially Maps to a QTL for FHB Resistance

Sequences for markers harboring QTLs for Fusarium Head Blight resistance were obtained from GrainGenes database and a BLAST analysis was developed to map sequences on wheat chromosomes using the IGWSC CSS v2 assembly for *T. aestivum*. Among the three homeologous genes, only the *Traes_2BS_14CA35D5D* gene was found co-located with a QTL responsible for FHB incidence and narrow opening of flowers between markers *Xbarc200* and *Xgwm210* surrounding this QTL (Gilsinger et al., 2005).

### The 2BS and 2DS Homeologous Genes Are Induced Following Infection by a DON-Producing *F. graminearum* Strain

The three identified homeologous wheat genes identified as potential orthologs of *the B. distachyon Bradi5g03300* gene were then studied at the transcriptional level. In order to reinforce their potential involvement in DON detoxification, the expression level of genes *Traes_2AS_99FF5CO43, Traes_2BS_14CA35D5D*, and *Traes_2DS_5CEOA969D* was determined following inoculation with two different strains of *F. graminearum*: *Fg*DON^+^ able to produce DON and *Fg*DON^−^ not able to produce DON (Cuzick et al., 2008). Using the SPADS software (Specific Primers and Amplicon Design Software, saruman.versailles.inra.fr), primer pairs specific for each gene were designed for a RT-qPCR application and were validated using nullisomic lines (Supplementary Table 1 and Supplementary Figures 1A–C).

**Figure 3:**
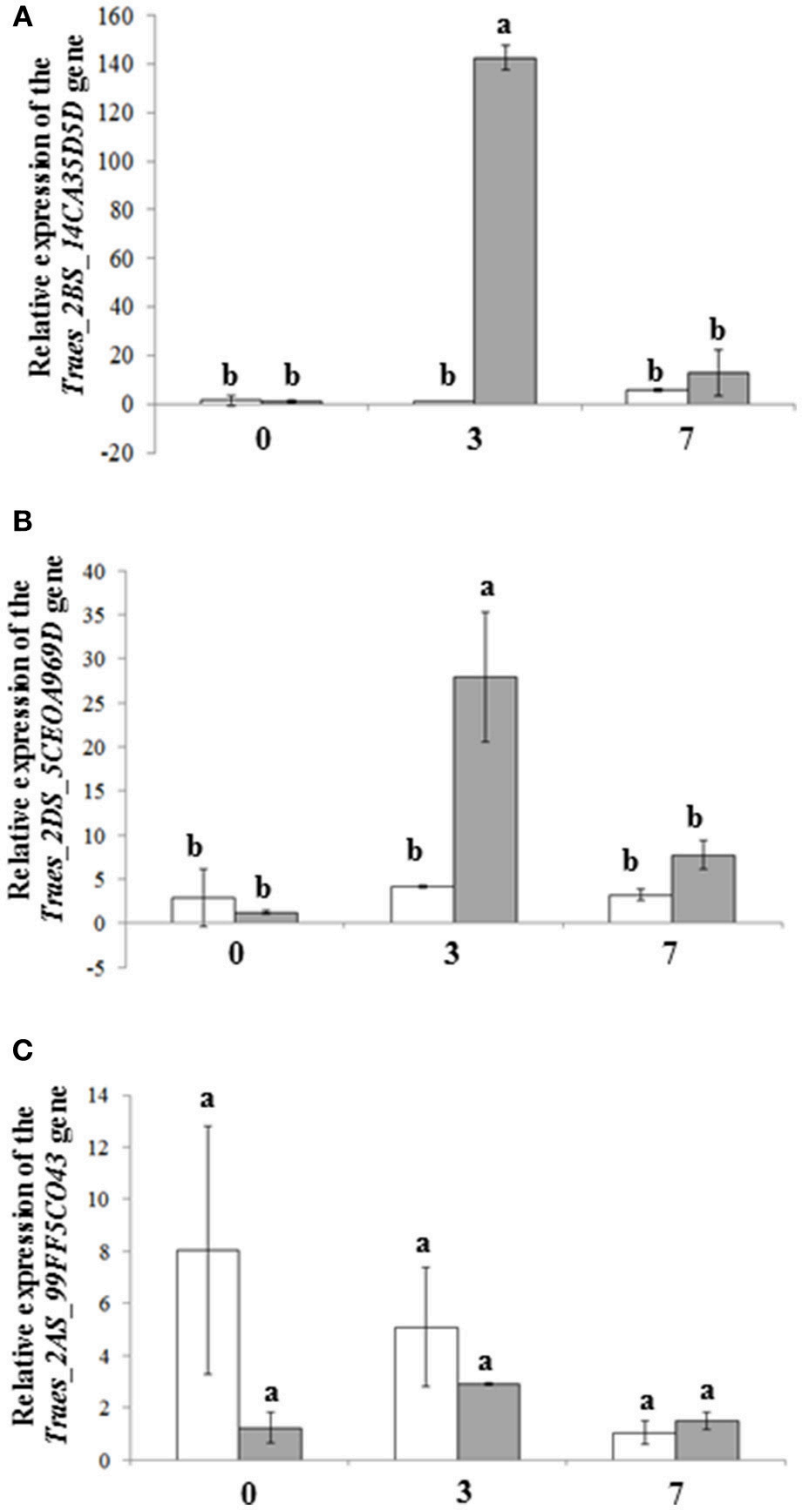
The *Traes_2BS_14CA35D5D* wheat gene is highly induced following infection by the *Fg*DON^+^ strain. Expression pattern of the *Triticum aestivum Traes_2BS_14CA35D5D* gene **(A)** and its homeologous genes *Traes_2DS_5CEOA969D* **(B)** *and Traes_2AS_99FF5CO43* **(C)**, 0, 3, and 7 days after inoculation (dai) by either *Fg*DON^+^ (gray bars), a *F. graminearum* strain able to produce deoxynivalenol (DON), and the *Fg*DON^−^ strain (white bars), which has lost the ability to produce deoxynivalenol (DON). The relative quantity of the gene transcripts as compared with the mock inoculation (Tween 20 0.01%) was calculated using the comparative cycle threshold method (ΔΔCt). *T. aestivum ACT* and *TUB* genes were used as endogenous reference genes to normalize the data for differences in input RNA between samples. Data represent mean values of three independent biological experiments, error bars represent the standard deviation. Different letters indicate significant differences between conditions; one way ANOVA and pairwise *t*-tests with Bonferroni correction, *p* < 0.05.

The expression of the *Traes_2BS_14CA35D5D* gene was highly induced (140-fold) in response to *F. graminearum* strain able to produce DON at 3 days after point inoculation (dai) compared with samples inoculated with the DON-deficient mutant (Figure 3A). Similarly, the *Traes_2DS_5CEOA969D* gene was induced 3 dai by the *Fg*DON^+^ strain but to a much lesser extent (nearly 5 times less than *Traes_2BS_14CA35D5D*, Figure 3B). For both genes, the specific induction by the *Fg*DON^+^ strain was no longer apparent at 7 dai No or a much weaker induction was detected following infection by the *Fg*DON^−^ strain. Neither significant induction nor differences were detected for the *Traes_2AS_99FF5C043* gene whatever the fungal strain considered (Figure 3C). We therefore focused on the *Traes_2BS_14CA35D5D* gene in the following experiments.

### *Brachypodium distachyon* Lines Expressing *Traes_2BS_14CA35D5D* Exhibit Increased Resistance to DON

A construction allowing the strong and constitutive expression of the best wheat candidate under the control of the *Z. mays Ubi1* promoter region was then generated (see section Materials and Methods and Supplementary Figure 2A). After selection and selfing, two independent transgenic lines, lines 1 and 2, were obtained carrying a single locus insertion of the construct and expressing the construct (Supplementary Figure 2B).

**Figure 4:**
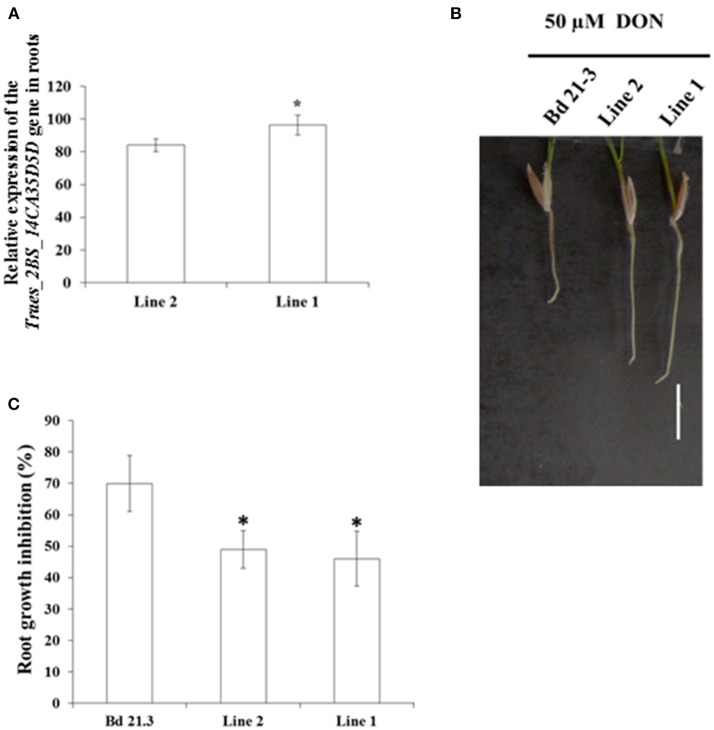
Expression of the *Traes_2BS_14CA35D5D* gene in transgenic *Brachypodium* lines confers root tolerance to DON. **(A)** Ratio of expression of the *Traes_2BS_14CA35D5D.1* gene in roots of the OE lines. The relative quantity of gene transcripts was calculated using: Ct (transgene)/Ct (control gene) *100. The *Brachypodium distachyon UBC18* gene was used as a reference to normalize the data for differences in input RNA between different samples. Data represent mean values of three independent biological experiments, error bars represent the standard deviation. Pairwise *t*-tests, *p* < 0.05. **(B)** Photograph showing typical root growth inhibition in Bd21-3, line 2, and line 1 by 50 μM DON measured on 7 days old seedling (Bar: 1 cm). **(C)** Root growth inhibition by 50 μM DON measured on the different lines. Data represent mean values of four independent biological experiments, *n* > 30 seeds per experiment, error bars represent the standard error, the asterisk indicates significant differences between Bd21-3 (control line) and each OE line: line 2 and line 1. One way ANOVA, *p* < 0.05.

**Figure 5:**
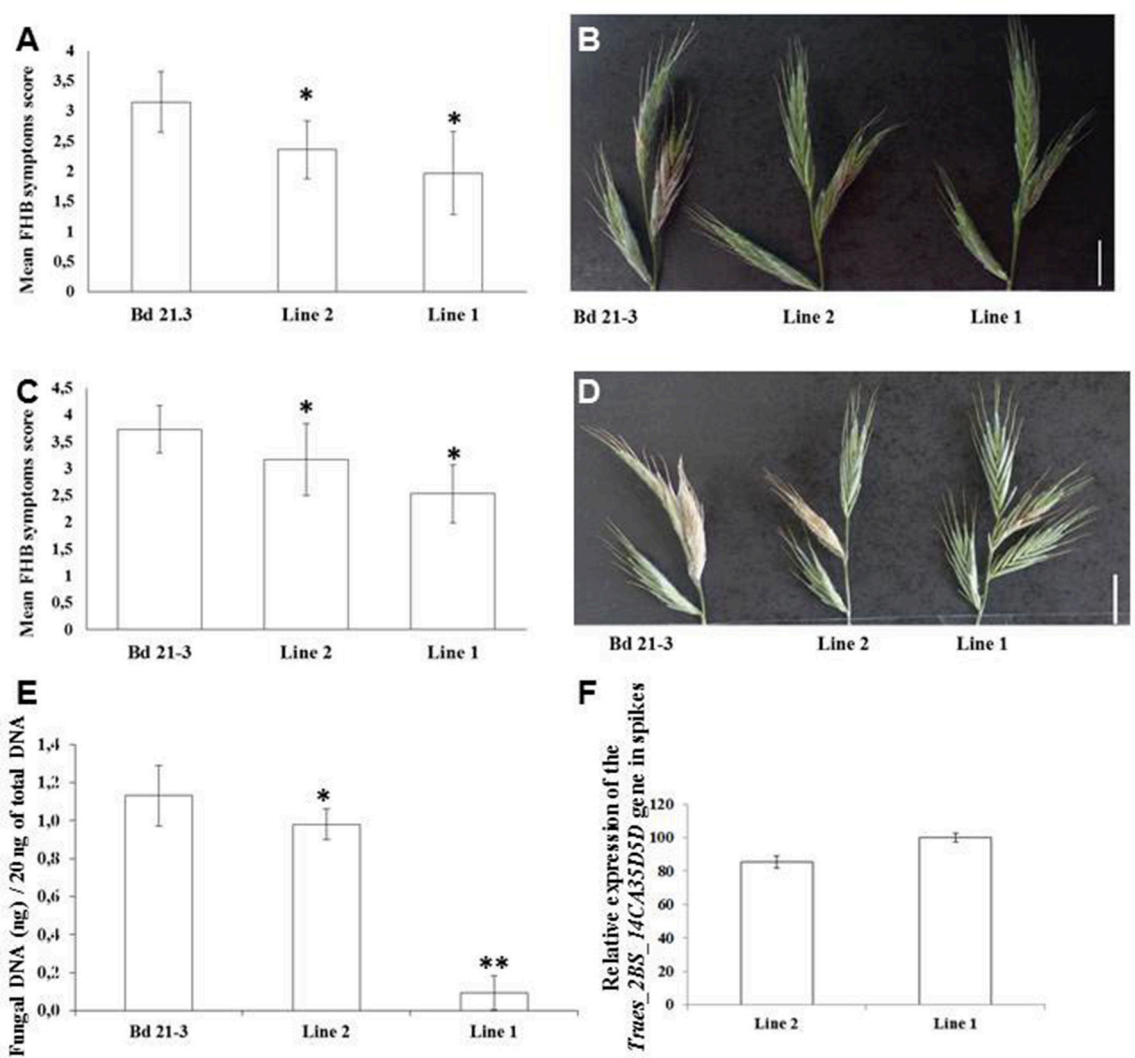
*Traes_2BS_14CA35D5D* contributes to resistance to *Fg* DON^+^ colonization in *Brachypodium distachyon* infected spikes. Percentage of spikelets exhibiting FHB symptoms on the inoculated spikes in *Brachypodium distachyon*, 7 **(A)** and 14 days **(C)** after point inoculation by the fungal strain *Fg* DON^+^ (according Scoring Scale). The asterisks indicate significant differences between Bd21-3 (control line) and each OE line; one way ANOVA, *p* < 0.05 (mean values of three independent biological replicates, *n* > 30 spikes per replicate). Photographs show representative symptoms on the different lines (transgenic and control) 7 days **(B)** and 14 days **(D)** after fungal inoculation by the *Fg*DON^+^ strain. **(E)** Quantification of fungal DNA by qPCR 14 days after point inoculation of *Brachypodium distachyon* spikes by the *Fg* DON^+^ strain. Data represent mean values of three independent biological experiments, error bars represent the standard deviation. Asterisks indicate significant differences between Bd21-3 (control line) and each transgenic line; pairwise *t*-tests, *p* < 0.005 (two asterisks) and *p* < 0.1 (one asterisk). **(F)** Ratio of expression of the *Traes_2BS_14CA35D5D.1* gene in spikes of the different transgenic lines. The relative quantity of gene transcripts was calculated using: Ct (transgene)/Ct (control gene) *100. The *Brachypodium distachyon UBC18* gene was used as the endogenous control to normalize the data for differences in input RNA between different samples. Data represent mean values of three independent biological replicates, error bars represent the standard deviation. Pairwise *t*-tests, *p* < 0.05.

We analyzed *Traes_2BS_14CA35D5D* relative expression in the two independent homozygous lines 1 and 2. RT-qPCR experiments were conducted to determine the expression of *Traes_2BS_14CA35D5D* in roots, spikes and leaves (Figures 4A, 5F and Supplementary Figure 3, respectively). Results showed that line 1 showed the highest *Traes_2BS_14CA35D5D* relative expression in all organs.

DON has already been reported to affect root growth in wheat (Masuda et al., 2007) and *B. distachyon* (Pasquet et al., 2016). To determine if *Traes_2BS_14CA35D5D* expression modifies root tolerance to DON, germinated seeds of the transgenic lines were transferred onto MS containing 50 μM DON in Petri dishes. While no difference of primary root development was observed between the transformed lines and Bd21-3 (control line) on agar medium without DON, strong phenotypes were observed on 50 μM DON after 7 days growth (Figure 4B). Root growth inhibition was around 46% for line 1, 48% for line 2 while it was around 69% for Bd21-3 (control) (Figure 4C, ANOVA, *p* < 0.05, *n* > 30). These results were in accordance with expression levels of the transgene in lines 1 and 2 (Figure 4A).

### Expression of *Traes_2BS_14CA35D5D* in *B. distachyon* Provides Resistance to Spike Colonization by *F. graminearum*

The transformed lines were further analyzed in a point inoculation assay on flowering plants using the *F. graminearum Fg*DON^+^ strain. The wild type line was used as a control. The FHB symptoms were scored 7 and 14 days after inoculation (dai) using a scale from 0 until 4 (in which 0 represents no symptoms and 4 a colonization extending the adjacent spikelet, Pasquet et al., 2016) mainly accounting for the ability of the fungal pathogen to colonize the spikelets and spikes. Scores obtained at 7 dai were 2.36 for line 2 and 1.97 for line 1, respectively, while the score was 3.15 for the wild type line Bd21-3 (ANOVA, *p* < 0.05, *n* > 30; Figures 5A,B). Average symptom scoring at 14 dai was 3.17 for line 2 and 2.53 for line 1 and 3.73 for the wild type line (ANOVA, *p* < 0.05, *n* > 30; Figures 5C,D). These results showed that transgenic *Brachypodium* lines carrying *Traes_2BS_14CA35D5D* exhibit increased resistance to FHB compared to the non-transformed control.

To assess the impact of *Traes_2BS_14CA35D5D* on fungal development more directly and quantitatively, fungal DNA was quantified by qPCR on the same point inoculated spikes of the *Brachypodium* wild-type line and transgenic lines. Fungal biomass was quantified at 14 days on lines 1, 2, and Bd21-3 (control). The results (Figure 5E) showed that 16 times less fungal DNA was detected in line 1 in comparison to Bd21-3. In line 2, there was 12 times less fungal DNA in comparison to the wild type line. Thus, the results on the fungal DNA abundance were in accordance with the previous phenotypical analysis. Quantitative resistance and reduced fungal development positively correlated to the transgene expression level (Figure 5F).

### Expression of *Traes_2BS_14CA35D5D* in *B. distachyon* Provides Resistance to Initial Infection by *F. graminearum*

**Figure 6:**
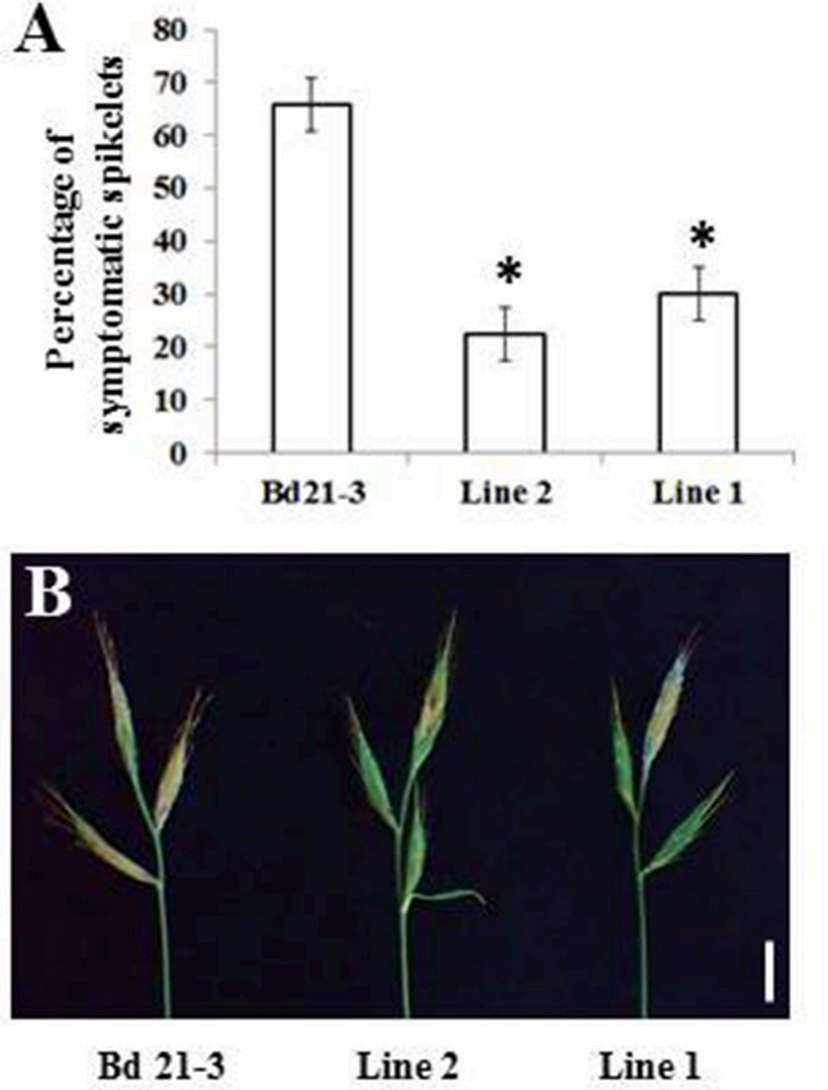
Expression of the *Traes_2BS_14CA35D5D* gene increases resistance to *F. graminearum* following spray inoculation. **(A)** Percentage of spikelets exhibiting FHB symptoms on half or more of the florets of the inoculated spikelet 7 days after spray inoculation by fungal strain *Fg* DON^+^ (mean values of three independent biological replicates, *n* > 30 spikes per replicate, error bars represent standard deviation, asterisks indicate significant differences between conditions; Newman and Keuls test, *p* ≤ 0.01). **(B)** Typical FHB symptoms observed on the transgenic lines or on the control line Apogee 7 days after spray inoculation by the *Fg* DON^+^ strain. Bar: 1 cm.

The previous results showed that transgenic *B. distachyon* lines expressing the wheat *UGT* gene exhibited higher resistance to fungal spike colonization compared to the control line. Spray inoculations were performed on the same plant lines to examine whether expression of *Traes_2BS_14CA35D5D* also conferred resistance to initial infection. Percentages of symptomatic spikelets were determined on both transgenic lines as well as the control line 14 days after spray inoculation. As shown in Figure 6, the transgenic lines 1 and 2 exhibited a strong reduction of the percentage of symptomatic spikelets at 14 dai, with 17 and 22%, respectively, as compared with the control line showing 66% of symptomatic spikelets (Figures 6A,B). Again, these results correlated well with the level of transgene expression in spikes of the two transgenic lines (Figure 5F).

### The Wheat UGT Encoded by the *Traes _2BS_14CA35D5D.1* Confers Resistance to Mycotoxin Accumulation in *B. distachyon*

**Figure 7:**
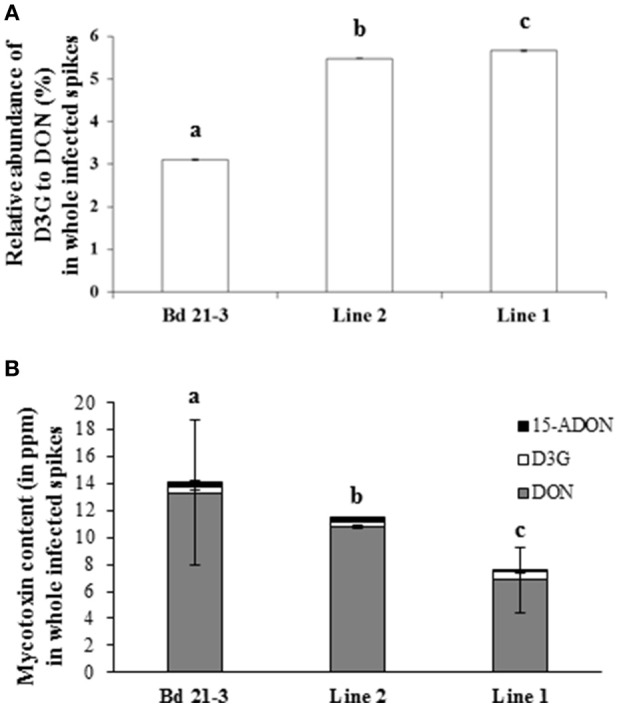
The wheat UGT encoded by the *Traes_2BS_14CA35D5D* gene glucosylates DON *in planta*. **(A)** Relative abundance of D3G to DON in whole spikes 14 days after inoculation (dai). **(B)** DON (gray bars), D3G (white bars), and 15-ADON (black bars) absolute quantification in whole spikes 14 dai. Data represent mean values of the independent biological replicates, error bars represent the standard deviation (different letters indicate significant differences between conditions: One way ANOVA and Duncan multiple range test (α = 0.01).

To determine if the enhanced resistance conferred by *Traes_2BS_14CA35D5D* expression was due to the increased glycosylation, we monitored DON, D3G and 15-ADON in infected spikes of the transgenic lines 1 and 2 and of the non-transgenic control line (Bd21-3). The relative abundance of D3G to DON was measured 14 dai in whole infected spikes in control and transformed lines. These values were 3.1% for Bd21-3, 5.4% for line 2, and 5.6% for line 1, therefore indicating a slight but significant increase in the transgenic lines (Figure 7A and Supplementary Figure 4). Absolute quantification of DON, D3G, and 15-ADON was performed at the same time (14 dai) in all lines. The quantity of total DON (DON+D3G+15-ADON) was reduced by 16 and 47% in lines 2 and 1, respectively, in comparison with the control (Bd21-3) (Figure 7B). However, the increase in D3G was low, with no significant difference for line 2 and only a 50% increase in D3 production in line 1, as compared with the wild-type line Bd21-3. Hence, expression of the *Traes_2BS_14CA35D5D* gene in *B. distachyon* leads to a decrease of total DON in infected spikes of the transgenic lines and to a slight increase of DON conjugation into D3 *in planta*.

## Discussion

Glucosylation of deoxynivalenol, catalyzed by plant UGTs, has been proposed as an interesting source of resistance to FHB in cereals (Gunupuru et al., 2017). However, only few studies could demonstrate that increased glucosylation of DON into D3G *in planta* conferred increased resistance to FHB. Expression of the barley Hv13248 UGT in wheat increased type II resistance both in controlled conditions and in field assays (Li et al., 2015). In *B. distachyon*, overexpression of the Bradi5g03300 led to increased resistance both to primary infection and to spike colonization (Pasquet et al., 2016). In wheat, a few UGT-encoding genes potentially involved in FHB resistance have been identified. The first ones, *TaUGT1* and *TaUGT2*, were only shown to be transcriptionally induced following either salt stress or *F. graminearum* infection (Lin et al., 2008). Using the Affymetrix wheat geneChip the *TaUGT3* gene encoding a putative DON resistance UGT, has been cloned and characterized from a wheat FHB resistant variety (Lulin et al., 2010). However, transgenic *Arabidopsis* lines carrying the *TaUGT3* gene did not exhibit clear tolerance to DON and could not be properly assessed for FHB resistance/susceptibility as Arabidopsis is not a host plant for this disease (Lulin et al., 2010). *TaUGT3* was further shown not to confer DON resistance in yeast (Schweiger et al., 2010). Nevertheless, a recent study showed that overexpression of this gene in wheat conferred quantitative type II resistance to FHB, most likely through the modification of expression of defense genes and not by glucosylation of DON (Xing et al., 2018). Using a heterologous system, the cDNA of another wheat candidate gene (*TaUGT12887*) associated with the locus *Qfhs.ifa-5A* for FHB resistance, was cloned and demonstrated its activity toward DON when expressed in yeast even if at a lower level than previously identified DON-detoxyfying UGTs (Schweiger et al., 2013b). An additional wheat UGT, encoded by the *TaUGT4* gene on chromosome 2D (Ma et al., 2015) was shown to be induced upon inoculation by *F. graminearum* or following DON treatment in Sumai 3 (resistant wheat cultivar) and Kenon 199 (susceptible wheat cultivar). However, this *UGT* gene was not further characterized. Finally, the *T. aestivum TaUGT5* gene was shown in a recent study to confer DON tolerance when overexpressed in *A. thaliana* and quantitative resistance to *F. graminearum*, both toward FHB and Crown Rot in field and controlled conditions, respectively, when overexpressed in wheat (Zhao et al., 2018). This study has not however investigated the ability of the TaUGT5 protein to conjugate DON into D3G. Therefore, it appears that both the criteria used for the identification of UGT-encoding candidate genes and the biological assays designed to investigate the potential of the candidates to detoxify DON and provide FHB resistance are essential parameters. In the present study, starting from a *UGT* gene previously shown to be involved in DON conjugation into D3G *in planta* (Pasquet et al., 2016), we developed an original synteny-based identification of wheat candidate genes and tested them *in planta* using the model cereal species *B. distachyon*.

### Identification and Characterization of a *Bradi5g03300* Wheat Ortholog

The introduction of *B. distachyon* as a cereal model has been particularly useful to navigate into the genome of the *Poaceae* family because of the high level of synteny between the genomes of *Brachypodium* and wheat (The International Brachypodium Initiative, 2010). In this study, we developed a synteny-based approach to identify wheat UGT-encoding genes potentially involved in DON glucosylation. Using *B. distachyon* model genes surrounding *Bradi5g03300* as baits, we identified the wheat syntenic region on the short arms of chromosomes 2A, 2B, and 2D. A more detailed micro-collinearity analysis revealed a complex situation with several events disrupting synteny. Moreover, BLAST analyses evidenced the existence of duplication events of UGT-encoding genes in both genomes leading to the presence of paralogous copies, either full-length genes or pseudogenes, on *B. distachyon* chromosome 5 as well as on at least one of wheat chromosomes 2 (see Supplementary Table 2), therefore rendering our analysis more complex. Such a situation has already been described for *UGT* genes in other plant species and was interpreted as indicative of recent gene duplications events which constitute a potential driving force for the recruitment of novel functions (Paquette et al., 2003; Yonekura-Sakakibara and Hanada, 2011). Nevertheless, application of thresholds and phylogenetic analysis allowed us to identify a triplet of wheat homeologous genes as potential wheat *Bradi5g03300* orthologs. Among these, the *Traes_2BS_14CA35D5D* gene was shown to be highly induced following wheat infection by a DON-producing strain of the fungal pathogen and to potentially co-localize with QTL responsible for FHB incidence. All together, these results favored this gene *Traes_2BS_14CA35D5D* as the wheat ortholog of the *B. distachyon Bradi5g03300* gene.

### Expression of *T. aestivum Traes_2BS_14CA35D5D* UGT- Encoding Gene in *B. distachyon* Confers Root Tolerance to DON and Quantitative Resistance to FHB

Phenotypic analyses were conducted on *B. distachyon* lines expressing the *Traes_2BS_14CA35D5D* cDNA. Transgenic lines showed increased root tolerance to DON with a positive correlation with the level of *Traes_2BS_14CA35D5D.1* cDNA expression. The same transgenic lines were also shown to exhibit higher resistance to FHB as compared to the control line. More precisely, we could demonstrate that the transgenic lines were more resistant to both initial infection and spike colonization, two distinct facets of FHB resistance frequently referred to as type I and type II resistance, respectively. Both aspects of quantitative resistance were also previously observed in *B. distachyon* transgenic lines overexpressing the Bradi5g03300 UGT-encoding gene (Pasquet et al., 2016). Few similar studies were conducted in wheat to test the impact of (over-)expression of UGT-encoding genes from barley (Li et al., 2015) or wheat (Xing et al., 2018; Zhao et al., 2018). Whatever the *UGT* gene tested, only type II resistance, namely resistance to spike/rachis colonization was observed. This discrepancy may point out specific differences between *B. distachyon* and *T. aestivum* spikes rather than gene-dependent differentials. Among these, the existence of extruding anthers which have been shown to be linked to susceptibility to FHB could be an important issue (Skinnes et al., 2010; Lu et al., 2013).

Transgenic *Brachypodium* lines over-expressing the *Traes_2BS_14CA35D5D* cDNA did not develop any sign of stress or developmental defect measured when growth parameters such as plant height, the number of tillers and of spikelets per spike were measured at BBCH65 (Supplementary Figure 5). These results may suggest that, as for the *B. distachyon* Bradi5g03300 protein, the wheat UGT does not play a major endogenous function. This rather may reflect a co-evolution between wheat and *F. graminearum* in which *Traes_2BS_14CA35D5D.1* may have evolved as a detoxification gene adapted to conjugate DON.

### The *Traes_2BS_14CA35D5D.1*-Encoded UGT Reduces Total DON Content *in Planta* Following *F. graminearum* Infection

Introgressing FHB resistance into commercial varieties cultivars results in a diminution of the content of both DON and its masked fraction, DON-3-*O-*glucose (D3G) correlated with type III resistance (Audenaert et al., 2013). The overexpression of the barley *Hv13248* gene in wheat has been shown to increase DON conjugation into D3G (Li et al., 2015) following direct application of DON into the spikes. In this study, we showed that expression of the wheat *Traes_2BS_14CA35D5D* gene in *B. distachyon* leads to a slight but significant increase of the relative abundance of D3G to DON in the line exhibiting the highest level of transgene expression. Nevertheless, the most obvious effect was observed on total DON accumulation with a nearly 50% reduction of overall mycotoxin content as compared with the control line. Even if direct comparison is difficult, *B. distachyon* lines overexpressing the *Bradi5g03300* gene also exhibited a strong reduction in total DON content but this reduction was around 75% as compared with the wild-type line Bd21-3 (Pasquet et al., 2016), with similar transgene expression levels (data not shown). As for the root growth assay, this result illustrates that the wheat UGT is most likely not as efficient as the *Brachypodium* one. However, in our previous work, impact on DON glucosylation was more obvious in earlier infection time-points such as 48 h after inoculation (Pasquet et al., 2016). Further experiments such as of the quantification of DON and its derivatives at earlier time points of infection are needed to better estimate the comparative efficacy of the two UGTs. Alternatively, our results could be explained by the involvement of the wheat UGT in quantitative resistance to fungal infection rather than in DON glucosylation *per se*. Further experiments, notably using a recombinant protein are needed to fully decipher the role of the wheat UGT during infection by *F. graminearum*.

The Bradi5g03300 UGT has recently been shown to be able to conjugate different mycotoxins belonging to trichothecenes, either type A or type B, in recombinant protein assays (Michlmayr et al., 2018). Future studies investigating whether this novel wheat UGT also exhibits a level of substrate flexibility will be of special interest, in particular to estimate its ability to be used as a source a resistance against a wider range of *Fusarium* sp. associated with FHB.

## Author Contributions

MG, FG, TL, and MD conceived and designed the experiments. FC conceived and designed synteny analyses. MG, FC, CM, FG, and MD conducted the experiments and analyses. MG, FC, and MD drafted the manuscript. All authors have read and approved the final version of the manuscript.

### Conflict of Interest Statement

The authors declare that the research was conducted in the absence of any commercial or financial relationships that could be construed as a potential conflict of interest.

## Supplementary Material

Supplementary Table 1 List of primers used in RT-qPCR and qPCR experiments.

Supplementary Table 2 Synteny analysis between B. distachyon proteins Bradi5g00230.1 to Bradi5g09987.1 and wheat deduced protein sequences.

Supplementary Presentation 1 Amino-acid sequences and multiple sequence alignement of the three homeologous wheat proteins considered as potential orthologs of the *B. distachyon* Bradi5g03300 UGT.

Supplementary Figure 1 Validation of the homeologous gene-specific primer pairs on nullisomic lines. Genomic DNA was extracted from the leaves of CS, Apogee, nullisomic lines and used as a template for PCR that was performed for 40 cycles under the following conditions: denaturation 94°C, 30 s; annealing 60°C, 30 s (RT-qPCR condition); extension 72°C, 30 s. **(A)** *Traes_2BS_TGACv1_14CA35D5D* (120 bp); **(B)** *Traes_2DS_5CE0A969D* (134 bp); **(C)** *Traes_2AS_99FF5C043* (126 bp).

Supplementary Figure 2 Transgene construct **(A)** and expression in *B. distachyon* transgenic lines 1 and 2 **(B)**. **(A)** Construction used in *Agrobacterium tumefasciens*-mediated transformation experiments. RB, LB: right and left borders of the T-DNA, respectively; *Zm, Zea mays*; HptR, hygromycin phosphotranferase-encoding gene conferring hygromycin B resistance. **(B)** The controls are a water (DNA-free) control and genomic DNA of non-transformed *Brachypodium distachyon* Bd21-3. As a positive control, the pIKb002 binary vector carrying the *Traes_2BS_14CA35D5D.1* cDNA was used. *2BS_1F* and *2BS_1R* are the primers used to amplify 120-bp PCR product specific for the *Traes_2BS_14CA35D5D.1* cDNA.

Supplementary Figure 3 Relative expression of the *Traes_2BS_14CA35D5D.1* cDNA gene in leaves of the *B. distachyon* transgenic lines 1 and 2. The relative quantity of gene transcripts was calculated using: Ct (transgene)/Ct (control gene) ^*^100. The *Brachypodium distachyon UBC18* gene was used as endogenous control to normalize the data for differences in input RNA between different samples. Data represent mean values of three independent biological replicates, error bars represent the standard deviation. Pairwise *t*-tests, *p* < 0.05.

Supplementary Figure 4 Chromatograms obtained on lines Bd21-3 **(A)**, 1 **(B)**, and 2 **(C)**. DON and its metabolites were extracted and analyzed on spikes 14 days after spray inoculation by fungal strain *Fg* DON^+^. Retention times are indicated in minutes.

Supplementary Figure 5 Phenotypic analyses of transgenic lines expressing the wheat *Traes_2BS_14CA35D5D.1* cDNA as compared with the wild-type *Brachypodium* Bd21-3 at BBCH65. **(A)** Plant height (cm) **(B)** Tillers number **(C)** Spikelets number. Data represent mean values of three independent experiments, error bars represent the standard deviation. One way ANOVA and pairwise *t*-tests, *p* < 0.05. No differences were observed between Bd21-3 (control line) and any of the two transgenic lines at BBCH65 stage, pairwise *t*-tests, *p* < 0.05.
