# Supplementary material for: Identification, Molecular Cloning, and Functional Characterization of a Wheat UDP-Glucosyltransferase Involved in Resistance to Fusarium Head Blight and to Mycotoxin Accumulation
Source: Front Plant Sci. 2018 Dec 13;9:1853. doi: 10.3389/fpls.2018.01853 (PMC6300724; doi:10.3389/fpls.2018.01853)
Supplement: Supplementary Table 1 — List of primers used in RT-qPCR and qPCR experiments. [file Table_1.DOCX]

**Supplementary Table 1.** List of primers used in RT-qPCR and qPCR experiments.

| **Gene** | **Nucleotide sequence** | **Polarity** | **Putative function** | **Use** |
| --- | --- | --- | --- | --- |
| *2AS* gene | 5’-TGCCATCGTTTTACCTTG-3’ | Forward | UDP-glycosyltransferase (putative) | RT-qPCR |
|  | 5’-CGAGCACAACAGAGGAGA-3’ | Reverse |  |  |
| *2BS* gene | 5’-GCGGTTGTTTGTGGTGTA-3’ | Forward | UDP-glycosyltransferase (putative) | RT-qPCR |
|  | 5’-TTCACTTTCGCTCTTCTGC-3’ | Reverse |  |  |
| *2DS* gene | 5’-CAAGAACAAAGCATCTCCTC-3’ | Forward | UDP-glycosyltransferase (putative) | RT-qPCR |
|  | 5’-TTGGACCTAACAACCCATAG-3’ | Reverse |  |  |
| *2BS* gene | 5’-ATGGAGAGCACGGGCCATGGAGG-3’ | Forward | UDP-glycosyltransferase (putative) | cDNA amplification |
|  | 5’- TCAAATTGACGAATACTTGGCAGCGAATTC -3’ | Reverse |  |  |
| *2BS* gene | 5’-CGCGGATCCGCGATGGAGAGCACGGG-3’* | Forward | UDP-glycosyltransferase (putative) | Transgene construct |
|  | 5’-GGATATCCTCAAATTGACGAATACTTGGCAGC-3’* | Reverse |  |  |
| *ACT* gene | 5’-TCTGGATCGGTGGCTCTATT-3’  5’-GCACTTCATGTGGACAATGC-3’ | Forward  Reverse | *T. aestivum* Actin | *T. aestivum* reference gene |
| *TUB* gene | 5'-GAGTTCACTGAGGCCGAGAG-3'  5’-GCTCCAGCTCTTCCTCTTC-3' | Forward  Reverse | *T. aestivum* Tubulin | *T. aestivum* reference gene |
| *Bradi4g00660* | 5'-ACCCTCTACGCTGGTGAGAC-3' | Forward | *B. distachyon* UBC18^a^ | *B. distachyon* reference gene |
|  | 5'-TTGCTGTAAATGTGCGGATG-3' | Reverse |  |  |
| 18S *F. graminearum* | 5'-GTCCGGCCGGGCCTTTCC-3' | Forward | Ribosomal region 18S^b^ (fungal reference gene) | qPCR (fungal reference gene) |
|  | 5'-AAGTCCTGTTTCCCCGCCACGC-3' | Reverse |  |  |

* Restriction sites used for the construct are underlined

^a^ Pasquet et al. 2014

^b^ Mudge et al. 2006
