# Supplementary material for: Identification, Molecular Cloning, and Functional Characterization of a Wheat UDP-Glucosyltransferase Involved in Resistance to Fusarium Head Blight and to Mycotoxin Accumulation
Source: Front Plant Sci. 2018 Dec 13;9:1853. doi: 10.3389/fpls.2018.01853 (PMC6300724; doi:10.3389/fpls.2018.01853)
Supplement: Supplementary Presentation 1 — Amino-acid sequences and multiple sequence alignement of the three homeologous wheat proteins considered as potential orthologs of the B. distachyon Bradi5g03300 UGT. [file Presentation_1.PPTX]

## Slide 1
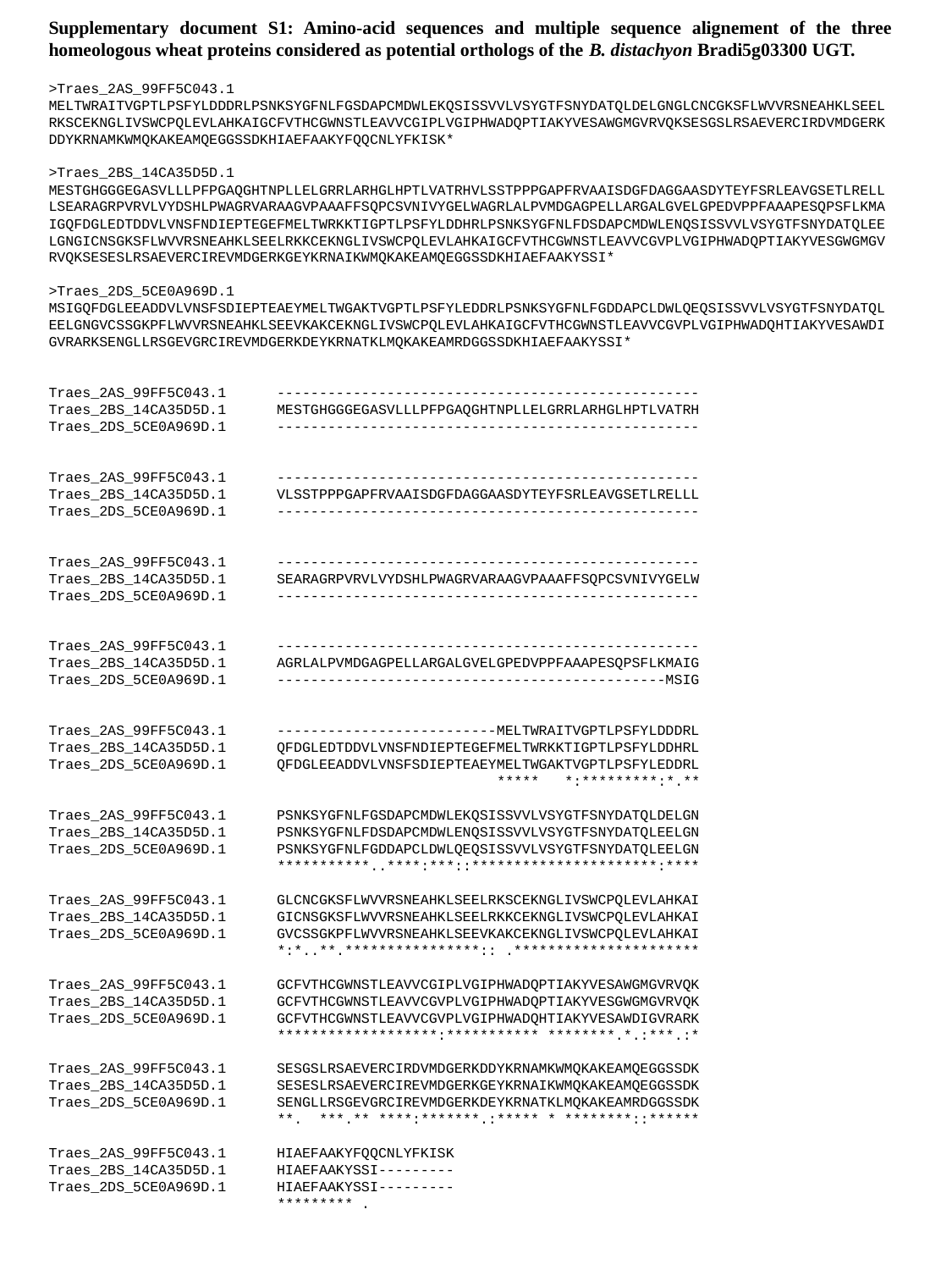

Supplementary document S1: Amino-acid sequences and multiple sequence alignement of the three homeologous wheat proteins considered as potential orthologs of the B. distachyon Bradi5g03300 UGT.
>Traes_2AS_99FF5C043.1
MELTWRAITVGPTLPSFYLDDDRLPSNKSYGFNLFGSDAPCMDWLEKQSISSVVLVSYGTFSNYDATQLDELGNGLCNCGKSFLWVVRSNEAHKLSEELRKSCEKNGLIVSWCPQLEVLAHKAIGCFVTHCGWNSTLEAVVCGIPLVGIPHWADQPTIAKYVESAWGMGVRVQKSESGSLRSAEVERCIRDVMDGERKDDYKRNAMKWMQKAKEAMQEGGSSDKHIAEFAAKYFQQCNLYFKISK*
>Traes_2BS_14CA35D5D.1
MESTGHGGGEGASVLLLPFPGAQGHTNPLLELGRRLARHGLHPTLVATRHVLSSTPPPGAPFRVAAISDGFDAGGAASDYTEYFSRLEAVGSETLRELLLSEARAGRPVRVLVYDSHLPWAGRVARAAGVPAAAFFSQPCSVNIVYGELWAGRLALPVMDGAGPELLARGALGVELGPEDVPPFAAAPESQPSFLKMAIGQFDGLEDTDDVLVNSFNDIEPTEGEFMELTWRKKTIGPTLPSFYLDDHRLPSNKSYGFNLFDSDAPCMDWLENQSISSVVLVSYGTFSNYDATQLEELGNGICNSGKSFLWVVRSNEAHKLSEELRKKCEKNGLIVSWCPQLEVLAHKAIGCFVTHCGWNSTLEAVVCGVPLVGIPHWADQPTIAKYVESGWGMGVRVQKSESESLRSAEVERCIREVMDGERKGEYKRNAIKWMQKAKEAMQEGGSSDKHIAEFAAKYSSI*
>Traes_2DS_5CE0A969D.1
MSIGQFDGLEEADDVLVNSFSDIEPTEAEYMELTWGAKTVGPTLPSFYLEDDRLPSNKSYGFNLFGDDAPCLDWLQEQSISSVVLVSYGTFSNYDATQLEELGNGVCSSGKPFLWVVRSNEAHKLSEEVKAKCEKNGLIVSWCPQLEVLAHKAIGCFVTHCGWNSTLEAVVCGVPLVGIPHWADQHTIAKYVESAWDIGVRARKSENGLLRSGEVGRCIREVMDGERKDEYKRNATKLMQKAKEAMRDGGSSDKHIAEFAAKYSSI*
Traes_2AS_99FF5C043.1 --------------------------------------------------
Traes_2BS_14CA35D5D.1 MESTGHGGGEGASVLLLPFPGAQGHTNPLLELGRRLARHGLHPTLVATRH
Traes_2DS_5CE0A969D.1 --------------------------------------------------
Traes_2AS_99FF5C043.1 --------------------------------------------------
Traes_2BS_14CA35D5D.1 VLSSTPPPGAPFRVAAISDGFDAGGAASDYTEYFSRLEAVGSETLRELLL
Traes_2DS_5CE0A969D.1 --------------------------------------------------
Traes_2AS_99FF5C043.1 --------------------------------------------------
Traes_2BS_14CA35D5D.1 SEARAGRPVRVLVYDSHLPWAGRVARAAGVPAAAFFSQPCSVNIVYGELW
Traes_2DS_5CE0A969D.1 --------------------------------------------------
Traes_2AS_99FF5C043.1 --------------------------------------------------
Traes_2BS_14CA35D5D.1 AGRLALPVMDGAGPELLARGALGVELGPEDVPPFAAAPESQPSFLKMAIG
Traes_2DS_5CE0A969D.1 ----------------------------------------------MSIG
Traes_2AS_99FF5C043.1 --------------------------MELTWRAITVGPTLPSFYLDDDRL
Traes_2BS_14CA35D5D.1 QFDGLEDTDDVLVNSFNDIEPTEGEFMELTWRKKTIGPTLPSFYLDDHRL
Traes_2DS_5CE0A969D.1 QFDGLEEADDVLVNSFSDIEPTEAEYMELTWGAKTVGPTLPSFYLEDDRL
 ***** *:*********:*.**
Traes_2AS_99FF5C043.1 PSNKSYGFNLFGSDAPCMDWLEKQSISSVVLVSYGTFSNYDATQLDELGN
Traes_2BS_14CA35D5D.1 PSNKSYGFNLFDSDAPCMDWLENQSISSVVLVSYGTFSNYDATQLEELGN
Traes_2DS_5CE0A969D.1 PSNKSYGFNLFGDDAPCLDWLQEQSISSVVLVSYGTFSNYDATQLEELGN
 ***********..****:***::**********************:****
Traes_2AS_99FF5C043.1 GLCNCGKSFLWVVRSNEAHKLSEELRKSCEKNGLIVSWCPQLEVLAHKAI
Traes_2BS_14CA35D5D.1 GICNSGKSFLWVVRSNEAHKLSEELRKKCEKNGLIVSWCPQLEVLAHKAI
Traes_2DS_5CE0A969D.1 GVCSSGKPFLWVVRSNEAHKLSEEVKAKCEKNGLIVSWCPQLEVLAHKAI
 *:*..**.****************:: .**********************
Traes_2AS_99FF5C043.1 GCFVTHCGWNSTLEAVVCGIPLVGIPHWADQPTIAKYVESAWGMGVRVQK
Traes_2BS_14CA35D5D.1 GCFVTHCGWNSTLEAVVCGVPLVGIPHWADQPTIAKYVESGWGMGVRVQK
Traes_2DS_5CE0A969D.1 GCFVTHCGWNSTLEAVVCGVPLVGIPHWADQHTIAKYVESAWDIGVRARK
 *******************:*********** ********.*.:***.:*
Traes_2AS_99FF5C043.1 SESGSLRSAEVERCIRDVMDGERKDDYKRNAMKWMQKAKEAMQEGGSSDK
Traes_2BS_14CA35D5D.1 SESESLRSAEVERCIREVMDGERKGEYKRNAIKWMQKAKEAMQEGGSSDK
Traes_2DS_5CE0A969D.1 SENGLLRSGEVGRCIREVMDGERKDEYKRNATKLMQKAKEAMRDGGSSDK
 **. ***.** ****:*******.:***** * ********::******
Traes_2AS_99FF5C043.1 HIAEFAAKYFQQCNLYFKISK
Traes_2BS_14CA35D5D.1 HIAEFAAKYSSI---------
Traes_2DS_5CE0A969D.1 HIAEFAAKYSSI---------
 ********* .
