# Supplementary figures and images for: Identification, Molecular Cloning, and Functional Characterization of a Wheat UDP-Glucosyltransferase Involved in Resistance to Fusarium Head Blight and to Mycotoxin Accumulation
Source: Front Plant Sci. 2018 Dec 13;9:1853. doi: 10.3389/fpls.2018.01853 (PMC6300724; doi:10.3389/fpls.2018.01853)

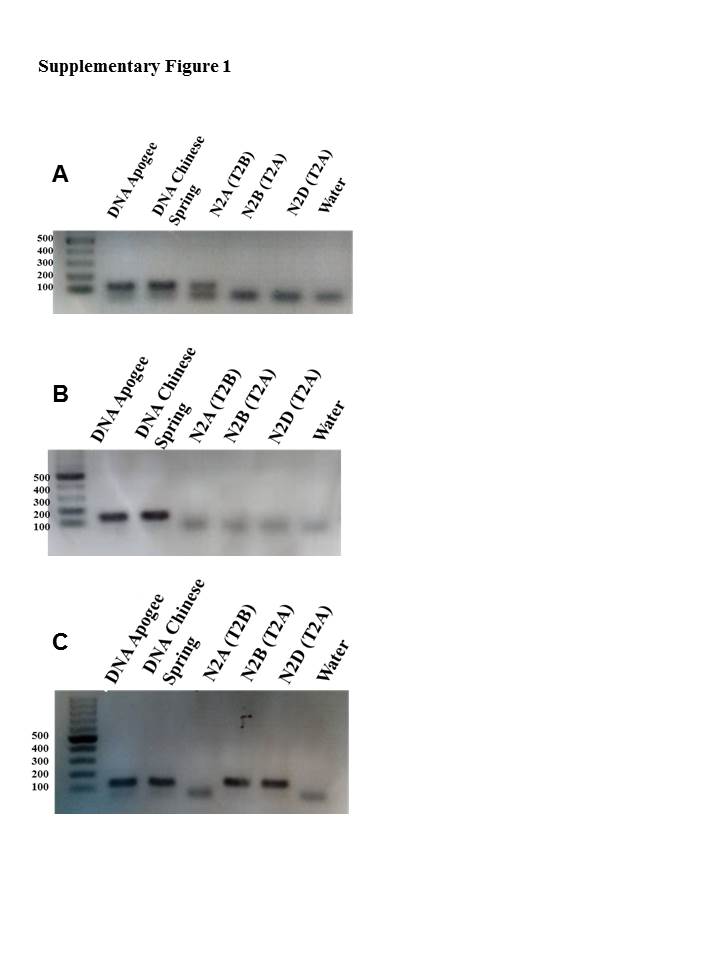

Supplement: Supplementary Figure 1 — Validation of the homeologous gene-specific primer pairs on nullisomic lines. Genomic DNA was extracted from the leaves of CS, Apogee, nullisomic lines and used as a template for PCR that was performed for 40 cycles under the following conditions: denaturation 94°C, 30 s; annealing 60°C, 30 s (RT-qPCR condition); extension 72°C, 30 s. (A) Traes_2BS_TGACv1_14CA35D5D (120 bp); (B) Traes_2DS_5CE0A969D (134 bp); (C) Traes_2AS_99FF5C043 (126 bp). [file Image_1.JPEG]

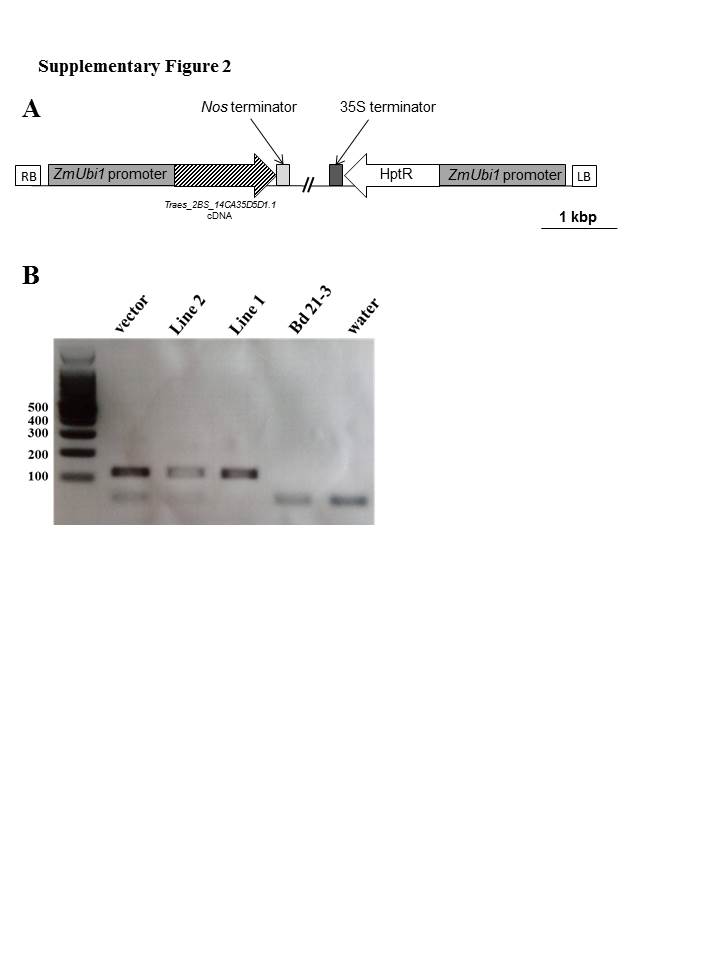

Supplement: Supplementary Figure 2 — Transgene construct (A) and expression in B. distachyon transgenic lines 1 and 2 (B). (A) Construction used in Agrobacterium tumefasciens-mediated transformation experiments. RB, LB: right and left borders of the T-DNA, respectively; Zm, Zea mays; HptR, hygromycin phosphotranferase-encoding gene conferring hygromycin B resistance. (B) The controls are a water (DNA-free) control and genomic DNA of non-transformed Brachypodium distachyon Bd21-3. As a positive control, the pIKb002 binary vector carrying the Traes_2BS_14CA35D5D.1 cDNA was used. 2BS_1F and 2BS_1R are the primers used to amplify 120-bp PCR product specific for the Traes_2BS_14CA35D5D.1 cDNA. [file Image_2.JPEG]

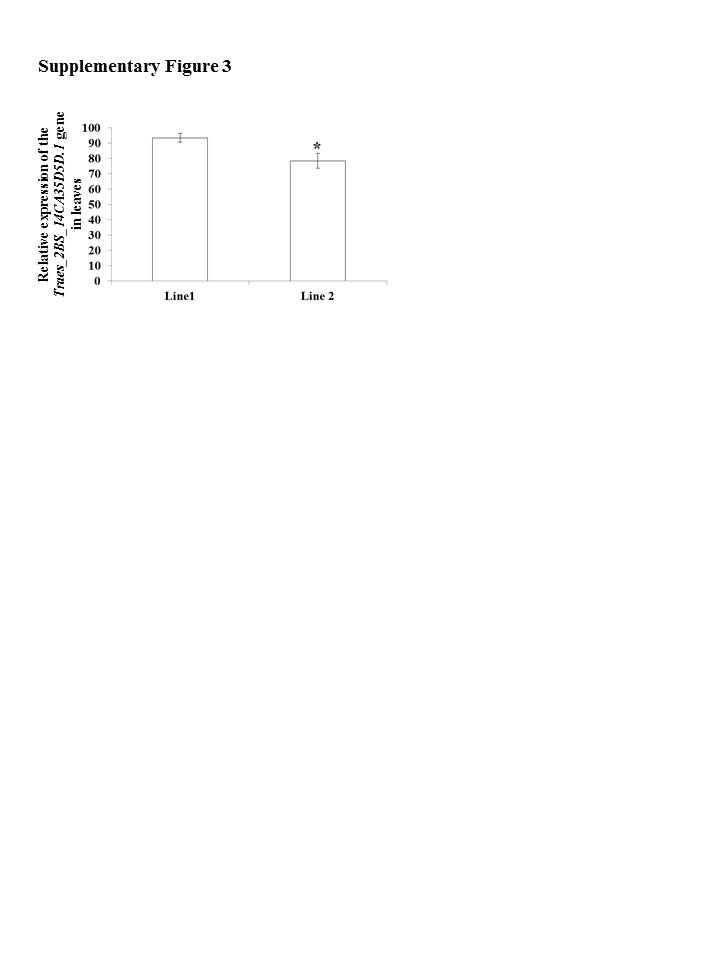

Supplement: Supplementary Figure 3 — Relative expression of the Traes_2BS_14CA35D5D.1 cDNA gene in leaves of the B. distachyon transgenic lines 1 and 2. The relative quantity of gene transcripts was calculated using: Ct (transgene)/Ct (control gene) *100. The Brachypodium distachyon UBC18 gene was used as endogenous control to normalize the data for differences in input RNA between different samples. Data represent mean values of three independent biological replicates, error bars represent the standard deviation. Pairwise t-tests, p < 0.05. [file Image_3.JPEG]

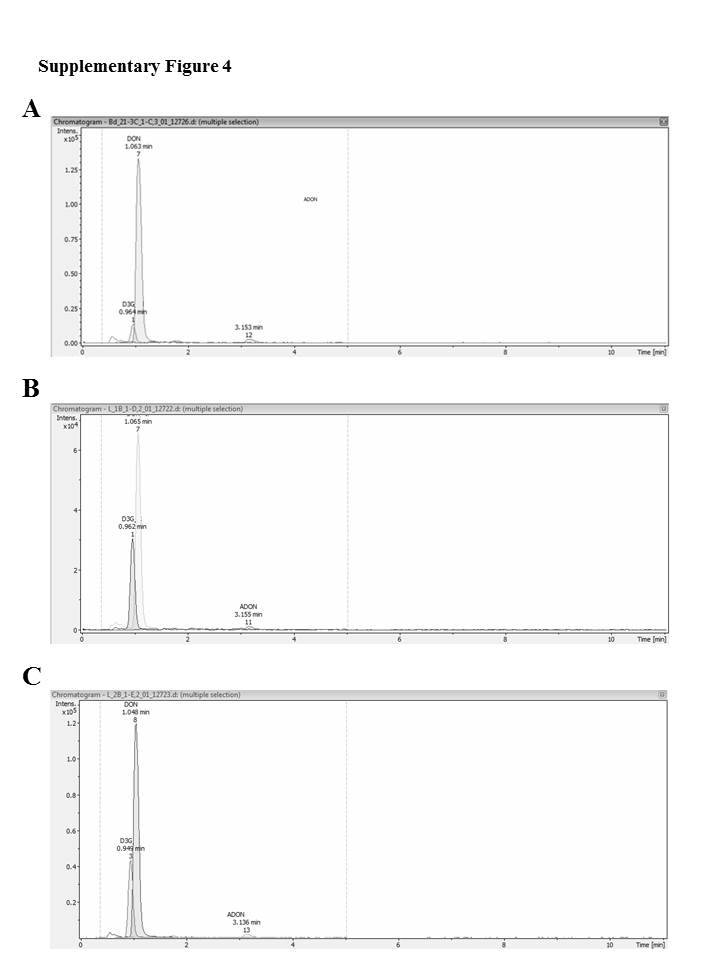

Supplement: Supplementary Figure 4 — Chromatograms obtained on lines Bd21-3 (A), 1 (B), and 2 (C). DON and its metabolites were extracted and analyzed on spikes 14 days after spray inoculation by fungal strain Fg DON+. Retention times are indicated in minutes. [file Image_4.JPEG]

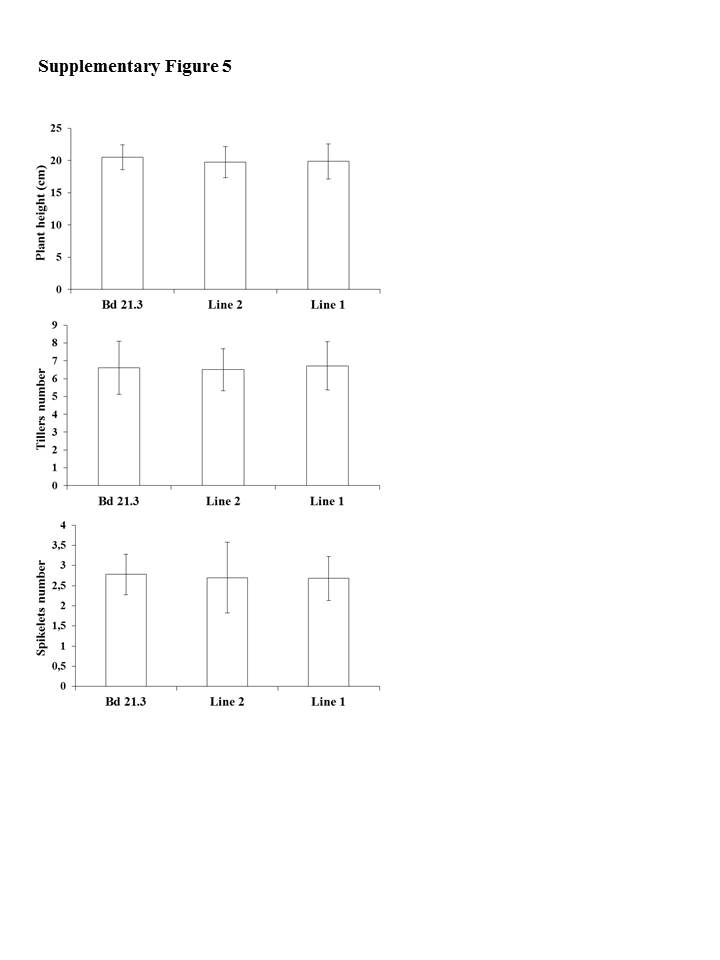

Supplement: Supplementary Figure 5 — Phenotypic analyses of transgenic lines expressing the wheat Traes_2BS_14CA35D5D.1 cDNA as compared with the wild-type Brachypodium Bd21-3 at BBCH65. (A) Plant height (cm) (B) Tillers number (C) Spikelets number. Data represent mean values of three independent experiments, error bars represent the standard deviation. One way ANOVA and pairwise t-tests, p < 0.05. No differences were observed between Bd21-3 (control line) and any of the two transgenic lines at BBCH65 stage, pairwise t-tests, p < 0.05. [file Image_5.JPEG]
